# Coronary arteries of the European bison (*Bison bonasus*)

**DOI:** 10.1186/s13028-015-0173-4

**Published:** 2015-11-25

**Authors:** Marta Kupczyńska, Karolina Barszcz, Katarzyna Olbrych, Michał Polguj, Grzegorz Wysiadecki, Mirosław Topol, Joanna Klećkowska-Nawrot

**Affiliations:** Department of Morphological Sciences, Faculty of Veterinary Medicine, Warsaw University of Life Sciences, SGGW, Nowoursynowska 159, 02-776 Warsaw, Poland; Department of Angiology, Interfaculty Chair of Anatomy and Histology, Medical University of Łódź, Narutowicza 60, 90-136 Lodz, Poland; Department of Normal and Clinical Anatomy, Interfaculty Chair of Anatomy and Histology, Medical University of Łódź, Narutowicza 60, 90-136 Lodz, Poland; Department of Animal Physiology and Biostructure, Faculty of Veterinary Medicine, Wroclaw University of Environmental and Life Sciences, Kożuchowska 1/3, 51-631 Wrocław, Poland

**Keywords:** Coronary arteries, Heart vascularization, European bison

## Abstract

**Background:**

The European bison (*Bison bonasus*) is an endangered species. More information on its anatomy is needed as only few studies have been published. This study is the first report on the morphology of the coronary vessels. Given the anatomical similarity between the European bison and other ruminants, the results of this study can be applied to other species, including endangered ones.

**Results:**

The study was conducted on 70 hearts of European bisons of both sexes, aged 5–20 years, with an average body weight of 449 kg. A distinct view of subepicardial arterial vessels was obtained by filling them with dyed synthetic latex (LBS 3060) and Plastogen G. There was a division of the common trunk of the left coronary artery into the interventricular paraconal branch and the left circumflex branch in 63 individuals (90 %). In five individuals (7.1 %), the presence of a third vessel, which was a branch of the interventricular septum, was observed. There was a lack of a common trunk in two individuals (2.9 %). Ramifications of the interventricular paraconal branch to the wall of the left ventricle were significantly larger than those to the wall of the right ventricle. In 17 individuals (24.3 %), the right coronary artery extended into the subsinuosal interventricular branch.

**Conclusion:**

The blood supply to the heart in bisons is provided by the left and right coronary arteries. In all the studied specimens, the left coronary artery was better developed than the right coronary artery.

## Background

Coronary vessels are well developed in mammals. They supply the myocardium with oxygen and nutritive elements. In humans, coronary arteries receive approximately 15 % of the total arterial blood. It is believed that this percentage is higher in animals and mainly depends on the individual’s health [1]. Typically, the mammalian heart is supplied with blood by the left coronary artery [*arteria* (*a.*) *coronaria sinistra*] and the right coronary artery (*a. coronaria dextra*) [1–3]. In humans, individuals with just a single coronary artery (SCA) have been reported [4]. The clinical significance of this variation is not clear but some autopsy studies indicate a possible link between SCA and sudden cardiac death. Turkmen et al. [4] based their research on the analysis of angiographic studies and determined the incidence of SCA to be 0.03 % (67 cases out of 215,140 individuals). Anomalies of one or both coronary ostia are present in approximately 0.6 % of humans [5]. In veterinary medicine, a similar variation has been observed in the lesser chinchilla (*Chinchilla lanigera*) [6].

Coronary vessels have been studied in a wide range of animals including Syrian hamster (*Mesocricetus auratus*) [7], guinea pig (*Cavia porcellus*) [8], the Angora rabbit [9], domestic dog (*Canis lupus f. domestica*) [10, 11], domestic cat [12, 13], donkey (*Equus asinus*) [14, 15], Angora and Akkamaran goats [16], roe deer (*Capreolus capreolus*) [17], Bactrian camel (*Camelus bactrianus*) [18], one-humped camel (*Camelus dromedarius*) [19], ringed seal (*Pusa hispida*) [20], porcupine (*Hystrix cristata*) [21, 22], shrew (*Suncus murinus*) [23], crab-eating macaque (*Macaca fascicularis*) [24, 25], grivet (*Cercopithecus aethiops*) [24] and rattlesnake (*Crotalus durissus*) [26]. There are also studies that refer to birds such as ostriches (*Struthio camelus*) [27] and chickens (*Gallus gallus domesticus*) [28]. However, the available literature provides no details on the topography of coronary arteries and their ramifications in the bison.

The aim of this study was therefore to investigate the morphology of the subepicardial vascularity of the European bison (*Bison bonasus*).

## Methods

The study was carried out on 70 hearts of European bisons of both sexes, aged 5–20 years, with an average body weight of 449 kg. The individuals examined were culled legally from the population of bisons in Białowieża Forest, Poland. The study was conducted with the permission of the Ministry of the Environment and the General Director for Environmental Protection in Poland.

The tissue surrounding the left and right coronary arteries was dissected in order to obtain access to the vessels.

Sixty-three hearts were dyed using synthetic LBS 3060 latex (Synthos Dwory Sp. z o.o, Poland). Those hearts were placed in a 10 % formalin for 6 weeks. The trunk of the left and right coronary artery and their ramifications were prepared [17, 29].

Corrosion casts were obtained from seven hearts using the multistage process to evaluate blood vessels. First, a 0.9 % NaCl solution was injected into the coronary arteries to flush out clots. Next, 20 ml of 3 % glutaraldehyde solution in a pH 7.4 cacodylate buffer was injected into the coronary arteries. The coronary arteries were then filled with Plastogen G (Plasto-Schmidt, Speyer, Germany) stained with green or red pigment and the heart was placed in water at 20 °C for 24 h to harden the resin. After hardening, the specimen was placed in a 40 % KOH solution at 50 °C for approximately 24 h to dissolve the organic tissue. The remnants of the dissolved tissue were removed from the specimen by continuous flushing with water for 38 h. The specimen was cleaned by a fast wash with warm water and a small amount of standard washing liquid, followed by a final flush with distilled water. The cast was later dried using airflow at room temperature for 2 days. The method was used with success in our previous studies [30, 31].

The specimens were examined morphologically using the ECLERIS (HALOLUX 150) surgical microscope with an integrated video channel. The diameters of the vessels were measured using software adapted for the metric analysis of images (AxioVision Rel. 4.7, Carl Zeiss MicroImaging GmbH, Jena, Germany). This software enables the visualization, archiving, processing and analysis of images, with particular emphasis on measurement functions. The terminology used in the manuscript is in accordance with prevailing veterinary nomenclature [32].

## Results

None of the animals included in the study had any pathological changes in the thoracic cavity.

### Morphology of the left coronary artery

**Fig. 1:**
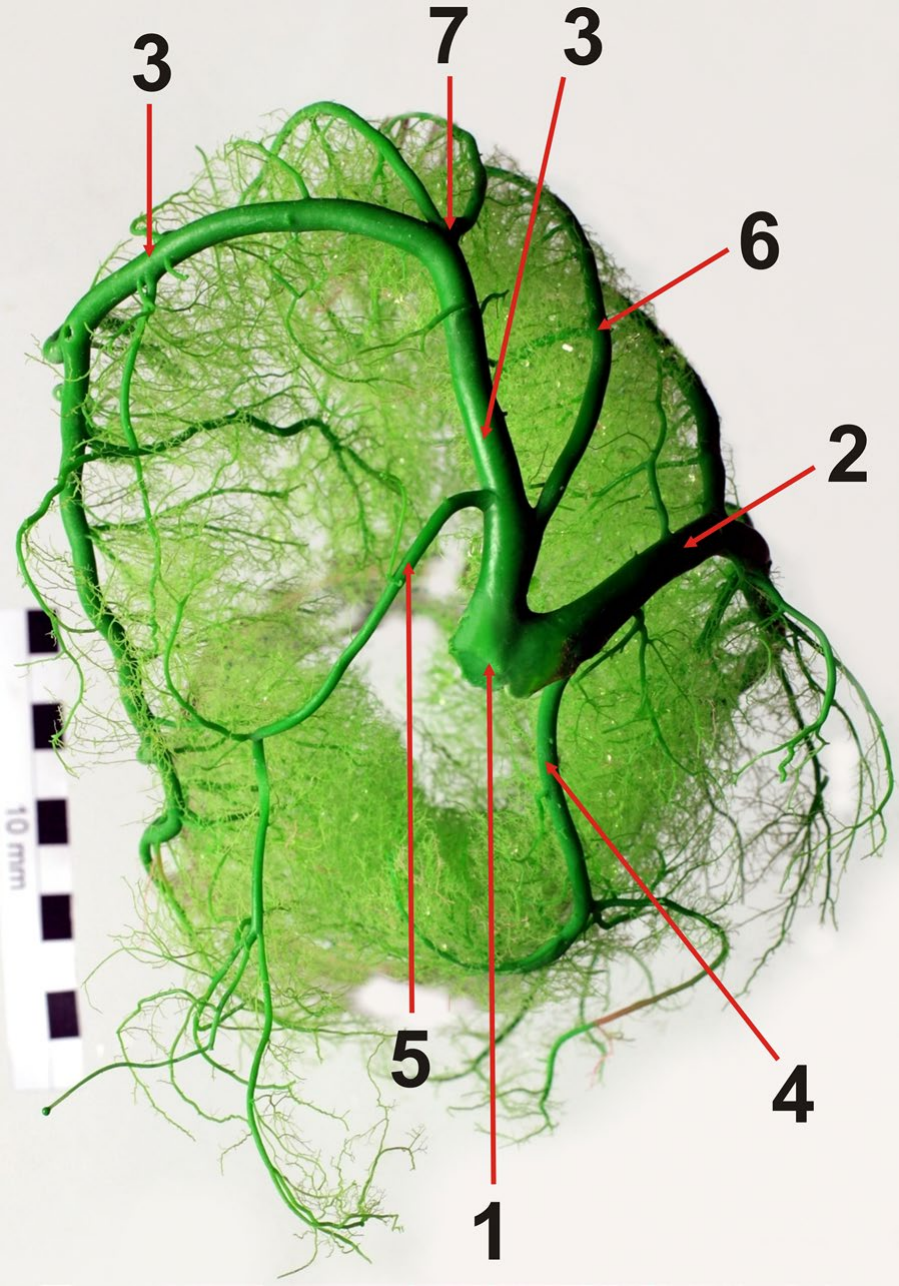
Branches of the left coronary artery. *1* the common trunk of the left coronary artery, *2* the paraconal interventricular branch (the descending part), *3* the left circumflex branch, *4* the left conal branch, *5* the proximal branch of the left atrium, *6* the proximal branch of the left ventricle and *7* the branch of the left ventricular border. Base of the heart; female, 7-years-old, body weight 430 kg

**Fig. 2:**
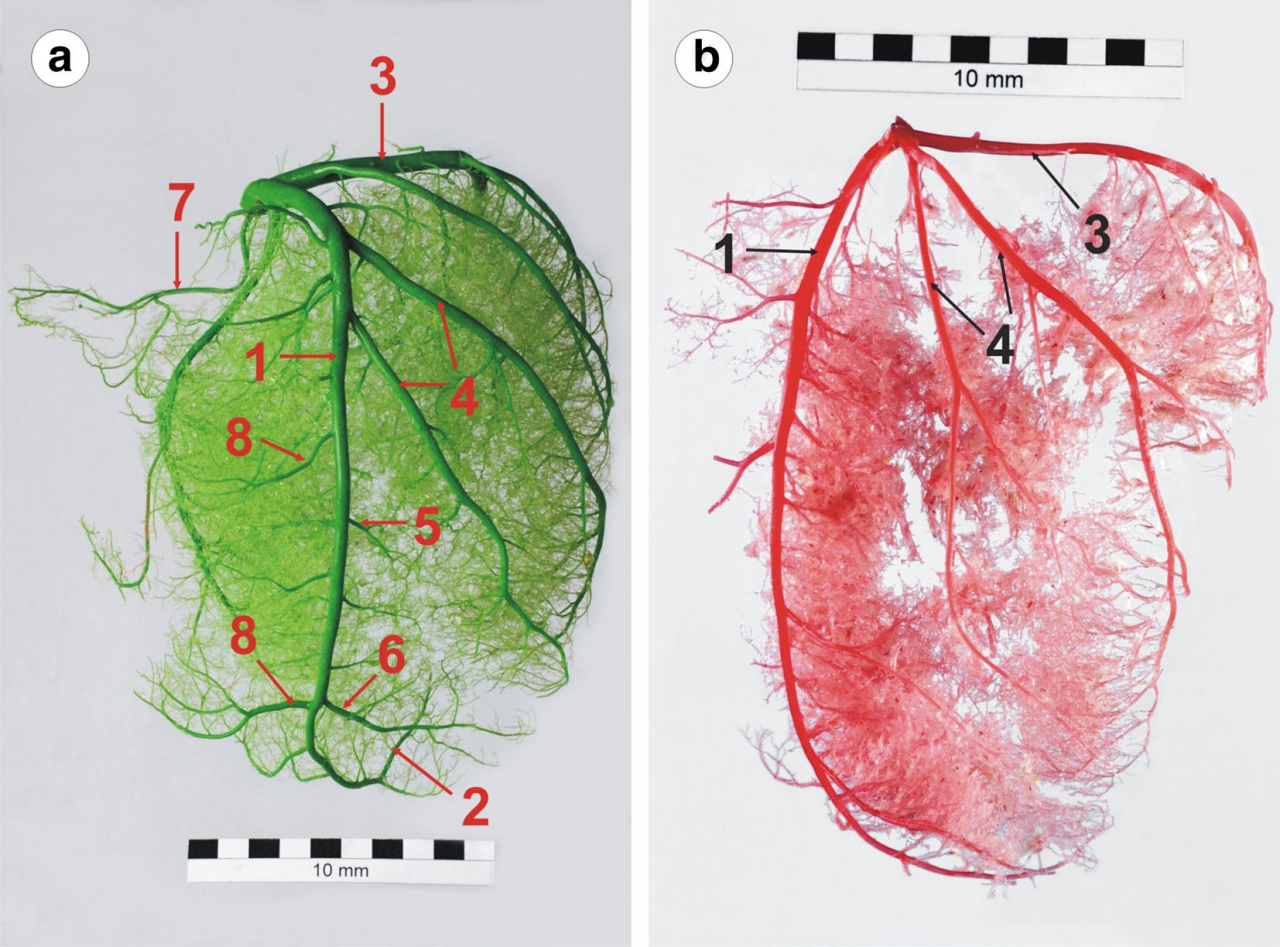
Branches of the left coronary artery (the auricular surface) in a European bison. **a** The separate origins of the proximal collateral branches (female, 7 years, 430 kg), **b** single proximal collateral branch (female, 15 years, 450 kg). *1* The paraconal interventricular branch (the descending part), *2* the paraconal interventricular branch (the ascending part), *3* the left circumflex branch, *4* the proximal collateral branches of the left ventricle, *5* the intermediate collateral branch of the left ventricle, *6* the distal collateral branch of the left ventricle, *7* the left conal branch and *8* the collateral branches of the right ventricle

The left coronary artery originated in the aortic sinus of the left semilunar cusp. The average vessel diameter was 15.7 mm. The short trunk (with an average length of 4.2 mm) of this vessel was placed between the left atrium and the initial segment of the pulmonary trunk. In 63 individuals (90 %), the common trunk divided into two vessels: a stronger interventricular paraconal branch and a weaker left circumflex branch (Figs. 1, 2). In 5 individuals (7.1 %), the presence of a third vessel being a branch of the interventricular septum was observed. The trunk can therefore be described as a trigeminal trunk. There was a lack of a common trunk in two individuals (2.9 %). In those cases, the interventricular paraconal branch and the left circumflex branch divided individually at the level of the aortic sinus of the left semilunar cusp.

The paraconal interventricular branch, *ramus* (*r.*) *interventricularis paraconalis*, was a highly developed vessel with an average diameter of 8.5 mm and was a direct extension of the left coronary artery. It ran towards the paraconal interventricular groove (in the descending part), and entered the subsinuosal interventricular groove above the notch of the cardiac apex (the ascending part) (Fig. 2a). The descending part of the paraconal interventricular branch divided into the (left) conal branch, the proximal collateral branches, the intermediate collateral branch, the distal collateral branch, the septal branches and numerous small ramifications to the lateral walls of the right and left ventricles on the auricular surface of the heart:.

The (left) conal branch, *r. coni arteriosi (sinister)*, emerged from the right wall of the stem vessel in all the studied bisons (Fig. 1). It either surrounded the arterial cone and ended at the right ventricular border, or it united with the terminal branches of the homonymous vessel derived from the right coronary artery.

The proximal collateral branches, *rami* (*rr*.) *collaterales proximales*, were the largest lateral vessels of the paraconal interventricular branch. In 31 individuals (44.3 %), two thick branches emerged from the initial segment of the stem vessel, separately or with a short common trunk (Fig. 2). They ran diagonally from the left ventricular wall to the left ventricular border. They then ran on the atrial surface of the heart where they divided into small vessels and penetrated the cardiac muscle. The proximal collateral branches generated small branches running along the wall of the left ventricle. In 26 individuals (37.1 %), the proximal collateral branches formed three large vessels. In 4 individuals (5.7 %), four large proximal collateral branches were present on the entire auricular surface of the left ventricle. In 9 cases (12.9 %), a single proximal collateral branch was observed, which ramified near its origin (Fig. 2b).

In most cases (55 individuals, 78.6 %), the intermediate collateral branch, *r. collateralis intermedius,* was poorly developed. It presented as a clear and strong vascular trunk in 15 individuals (21.4 %). It originated from the left wall of the paraconal interventricular branch, approximately half-way along the paraconal interventricular groove and ran along the wall of the left ventricle.

The distal collateral branch, *r. collateralis distalis,* ran from the left side of the stem vessel. Its ramifications reached the left ventricular border and supplied the walls of the left ventricle close to the cardiac apex.

There were numerous septal branches, *rr. septales*, along the entire course of the paraconal interventricular branch. They penetrated the interventricular septum where they gave off smaller branches.

Apart from the aforementioned thick vascular trunks, there were numerous small right ventricular branches, *rr. ventriculi dextri* and left ventricular branches, *rr. ventriculi sinistri,* which supplied the walls of both ventricles.

As described above, the ascending part, *pars ascendens* of the paraconal interventricular branch was a direct extension of the paraconal interventricular branch on the atrial surface of the heart. It proceeded dorsally from the notch of the cardiac apex in the subsinuosal interventricular groove. It gave off ramifications to lateral walls of both ventricles and the interventricular septum.

The left circumflex branch, *r. circumflexus sinister*, was the weakest ramification of the left coronary artery. It had a diameter of 5.7 mm measured in its proximal segment.

The circumflex branch initially ran in the coronary groove on the cardiac auricular surface, then passed the left ventricular border and reached the atrial surface of the heart. Most frequently (53 individuals, 75.7 %), it extended in the subsinuosal interventricular groove into the subsinuosal interventricular branch. Along its course, it gave off some small branches to the lateral wall of the left and right ventricle, and penetrated the cardiac muscle in the middle of the groove. In the remaining bisons (17 individuals, 24.3 %), the left circumflex branch ended with small ramifications. In those cases, the subsinuosal interventricular branch was supplied by the right coronary artery and ran in the subsinuosal interventricular groove. The circumflex branch was surrounded by large amounts of adipose tissue and was covered by the ventral border of the left atrial auricle on the auricular surface of the heart.

The following sub-branches emerged from the circumflex branch: the proximal branch of the left ventricle, the proximal branch of the left atrium, the branch of the left ventricular border, the intermediate branch of the left atrium, the distal branch of the left ventricle, the distal branch of the left atrium, the subsinuosal interventricular branch and numerous small ramifications to the lateral walls of the left atrium and the left ventricle of the heart.

**Fig. 3:**
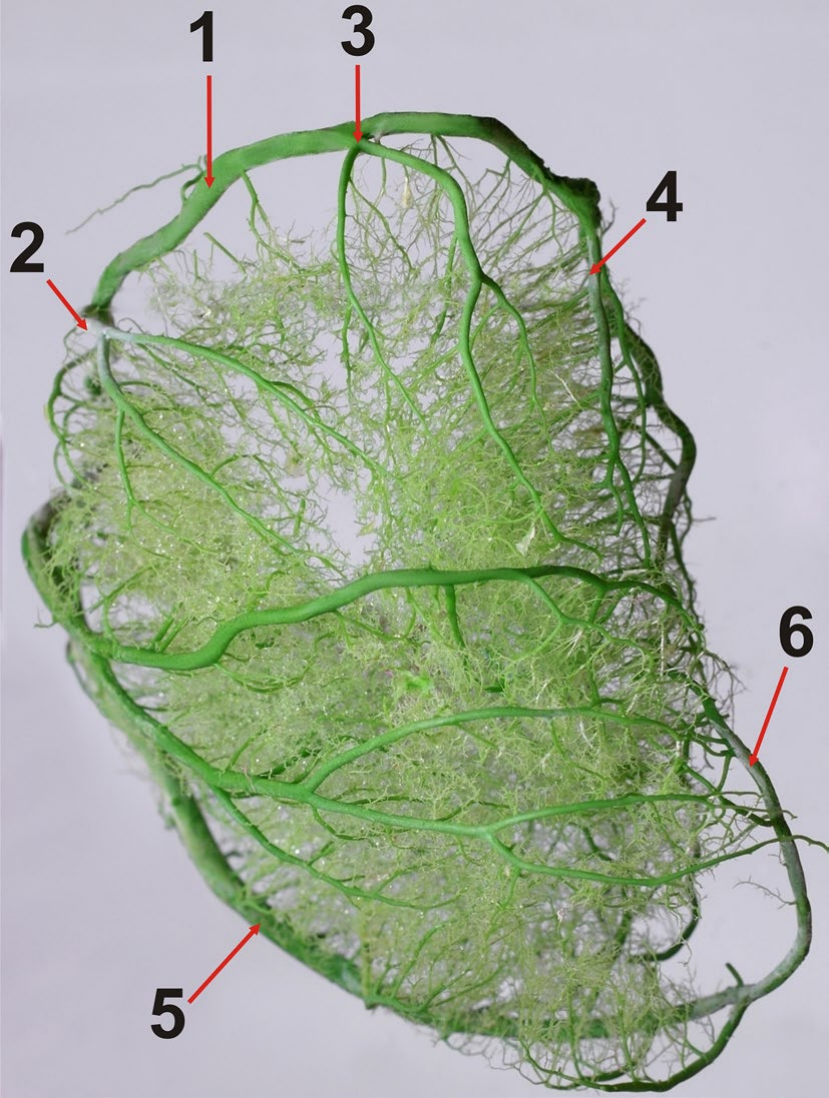
Branches of the left circumflex branch in a European bison. *1* the left circumflex branch, *2* the proximal branch of the left ventricle, *3* the branch of the left ventricular border, *4* the distal branch of the left ventricle, *5* the paraconal interventricular branch (the descending part), *6* the paraconal interventricular branch (the ascending part). Left ventricular border, male, 13-years-old, body weight 420 kg

The proximal branch of the left ventricle (*r. proximalis ventriculi sinistri)* was correctly developed in 17 individuals (24.3 %). It originated at the beginning of the (left) circumflex branch (Fig. 3), proceeded caudally and ventrally on the auricular surface of the left ventricle and reached the middle part of the left ventricular border, where it entered the atrial surface and ended as numerous ramifications in the lateral wall of the left ventricle or at the cardiac apex. It was poorly developed in 53 individuals (75.7 %).

The proximal branch of the left atrium, *r. proximalis atrii sinistri*, ran from the medial wall of the (left) circumflex branch towards the dorsal wall of the left atrium covered by a thin layer of cardiac muscle.

The left circumflex branch divided under the left auricle to form the branch of the left ventricular border, *r. marginis ventricularis sinistri* (Fig. 3). This vessel was clearly identifiable in all the individuals. It proceeded ventrally, along the left ventricular border and ended at half of its length. Along its course, that vessel gave off ramifications to the lateral wall of the left ventricle and the left ventricular border.

The intermediate branch of the left atrium, *r. intermedius atrii sinistri*, emerged immediately below the branch of the left ventricular border. It ran dorsally and slightly cranially across the coronary groove and entered the cavity between the left auricle and the left azygos vein.

The (left) circumflex branch gave off the distal branch of the left ventricle, *r. distalis ventriculi sinistri*, on the atrial surface (Fig. 3). That branch ran ventrally on the wall of the left ventricle, close to the left ventricular border. It ended in the middle of the left ventricle or slightly beneath the ventricular wall.

The distal branch of the left atrium, *r. distalis atrii sinistri*, was the least developed branch derived from the circumflex branch. It ran in a groove between the main caudal vein and the pulmonary veins.

Several small branches originating from the left circumflex branch and supplying the left ventricle and atrium, *rr. ventriculi sinistri*, *rr. atrii sinistri*, were observed.

### Morphology of the right coronary artery

**Fig. 4:**
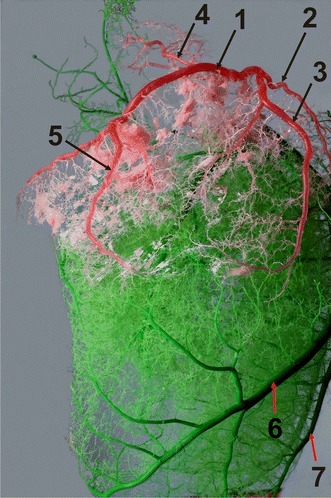
Branches of the right circumflex branch in a European bison. *1* the right circumflex branch, *2* the right conal branch, *3* the branch of the right ventricular border, *4* the intermediate branch of the right atrium, *5* the distal branch of the right ventricle, *6* the paraconal interventricular branch (the descending part) and *7* the intermediate collateral branch of the left ventricle. Right ventricular border; male, 8-years-old, body weight 570 kg

The right coronary artery, *a. coronaria dextra*, was found to be less developed than the left coronary artery. Its average diameter was 7.1 mm. It originated in the right coronary sinus of the aortic valve in all the subjects. At the base of the heart, it ran between the right atrium and the pulmonary trunk. It reached the right ventricular border, entered the coronary groove and was visible in some individuals as the right circumflex branch (*r. circumflexus dexter*) (Fig. 4). The initial section of the right coronary artery was covered by the auricle of the right atrium. It was surrounded by large amounts of adipose tissue along its entire length. Finally, the right coronary artery reached the subsinuosal interventricular groove, where it typically (53 individuals, 75.7 %) branched off a few small vessels. In the remaining animals (17 individuals, 24.3 %), the right coronary artery extended into the subsinuosal interventricular branch.

Numerous vessels branched off from the right coronary artery: the (right) conal branch, the proximal branch of the right atrium, the proximal branch of the right ventricle, the branch of the right ventricular border, the intermediate branch of the right atrium, the distal branch of the right ventricle and the distal branch of the right atrium.

The (right) conal branch, *r. coni arteriosi* (*dextra*), originated from the right wall of the right coronary artery at its orifice from the ascending aorta in all individuals and surrounded the arterial cone (Fig. 4). Multiple small vessels branched from the main trunk and ran towards the arterial cone.

The proximal branch of the right atrium, *r. proximalis atrii dextri*, was the largest vessel supplying the right atrium. It branched off at various levels of the right coronary artery, prior to the origin of the (right) conal branch. It ran on the right on the medial wall of the auricle of the right atrium, towards the opening of the cranial vena cava. The proximal branch of the right ventricle gave off small branches to the wall of the right atrium and the cranial vena cava.

The proximal branch of the right ventricle, *r. proximalis ventriculi dextri*, originated below the (right) conal branch and below the auricle of the right atrium. It ran towards the cardiac auricle on the wall of the right ventricle, slightly to the left of the right ventricular border and divided in the middle into two branches. The branch of the right ventricular border, *r. marginis ventricularis dextri* was a large vessel that ran along the right ventricular border and ended at various distances from the notch of the cardiac apex (Fig. 4). It mostly lied under the cardiac muscles, and only its initial short segment was surrounded by adipose tissue. The branch of the right ventricular border vascularized a vast part of the right ventricle in the proximity of the right ventricular border.

The intermediate branch of the right atrium, *r. intermedius atrii dextri*, was a small vessel that separated from the stem vessel at some distance from the branch of the right ventricular border (Fig. 4). It ran on the lateral wall of the right atrium, between its auricle and the cranial vena cava. This branch gave off vessels towards the auricle of the right atrium.

The distal branch of the right ventricle, *r. distalis ventriculi dextri*, originated in the distal segment of the right coronary artery on the atrial surface of the heart (Fig. 4). It ran along the lateral wall of the right ventricle, reaching the area near the subsinuosal interventricular groove. Along its course, it gave off several small branches to the lateral wall of the right ventricle.

The distal branch of the right atrium, *r. distalis atrii dextri*, was a very small vessel. After it stemmed from the right coronary artery in the proximity of the subsinuosal interventricular groove, it ran along the lateral wall of the right atrium and ramified near the opening of the coronary sinus and the caudal vena cava.

## Discussion

The heart of the European bison is vascularized by two well-developed (left and right) coronary arteries. The left coronary artery originated from the left coronary sinus of the aortic valve. In 63 bisons (90 %), the main trunk was divided into two terminal branches: the paraconal interventricular branch and the left circumflex branch. This type of trunk anatomy has also been reported in ringed seal [20], donkey [15], Bactrian camel [18] and roe deer [17]. Some reports indicate that the left coronary artery has three terminal vessels: the paraconal interventricular branch, the left circumflex branch and the interventricular septum branch [10, 29, 33].

There is no consensus as to the typical arrangement of the trunk of the left coronary artery in carnivores. Some authors suggest the bipartite type of trunk of the left coronary artery is the most common in dogs [10, 34, 35]. However, according to Blair [33], triple branching (tripartiate type) of the left coronary artery is typical in dogs. In cats, Vladova [13] only describes bipartite branching of the trunk. Meanwhile, observations by Barszcz et al. [29] showed bipartite branching of the trunk in 78.3 % and tripartite branching in 21.7 % of studied cats.

As previously mentioned, one of the terminal vessels of the left coronary artery is the left circumflex branch. In the studied European bisons, the left circumflex branch gave off branches to the wall of the left atrium and left ventricle. It was observed that the branches to the left atrium were significantly less developed than the branches to the walls of the left ventricle. Analogous observations have been made in dogs and cats [29, 35]. The largest ramification in the bisons was represented by the branch of the left ventricular border as in dogs, rabbits and cats [9, 29, 35].

The interventricular paraconal branch was the single, most strongly developed vessel, running along the cardiac auricular surface within the interventricular paraconal groove. The distal segment of the vessel passed onto the atrial surface of the heart. Due to the characteristic course of the main trunk of the discussed vessel, the descending and ascending parts could be distinguished. A similar course of the interventricular paraconal branch was reported in roe deer [17]. The interventricular paraconal branch in human anatomy is termed the anterior interventricular branch (*r. interventricularis anterior*) [3]. In humans, some rare cases of a double trunk of this vessel have been reported [36, 37].

The ramifications of the interventricular paraconal branch to the wall of the left ventricle were significantly larger than corresponding arteries supplying the wall of the right ventricle. Studies conducted in dogs and cats have shown that the number of collateral vessels varies between individuals and that they may occur as single, double or even triple homonymous ramifications [11, 29].

The presence of a single right coronary artery was observed in all the studied bisons. However, the available literature describes a rare condition in dogs with an additional right coronary artery (*a. coronaria dextra accessoria*). This vessel originates in the right aortic sinus of the aortic valve and runs very closely to the main right coronary artery [35]. The branches supplying the wall of the right atrium and the wall of the right ventricle originated in the trunk of this vessel. Similar observations were made in humans [38].

The origin of the subsinuosal interventricular branch is evident from many sources. It originates from the left coronary artery in dogs, cattle and roe deer [1, 17, 35]. However, in the pig, donkey, Bactrian camel and ringed seal, subsinuosal interventricular branch is an extension of the right coronary artery [1, 15, 18, 20]. In the majority of the studied bisons, the subsinuosal interventricular branch (in 53 individuals, 75.7 %) was a terminal vessel of the left circumflex branch. In 17 cases (24.3 %), the subsinuosal interventricular branch was an extension of the right coronary artery. Barszcz et al. [29] found three types of origins of subsinuosal interventricular branch in cats. Most frequently (45 %), the subsinuosal interventricular branch was an extension of the left circumflex branch. However, in approximately 40 % of cats, it was a direct continuation of the right circumflex branch. In 15 % of the cats, two strong trunks of the subsinuosal interventricular branch were observed, each departing from one or both circumflex branches. Observations by Vladova [13] showed that the subsinuosal interventricular branch was an extension of the left coronary artery in cats.

Two main types of vascularization of the atrial surface of the heart in the bisons were observed. In 53 cases (75.7 %), the subsinuosal interventricular branch was an extension of the left circumflex branch while in 17 cases (24.3 %), it was a direct continuation of the right circumflex branch.

## Conclusions

The blood supply of the heart in the European bison is provided by the left and right coronary arteries, with the left coronary artery being better developed than the right.
